# Heart Troubles & Cactus Grandiflora & Cimicifuga

**Published:** 1881-04

**Authors:** 


HEART TROUBLES, AND CACTUS GRANDIFLORA AND CIMICIFUGA.—It is undoubtedly a proper and useful plan to "try all things and hold fast to that which is good," in therapeutics as well as in other things. For a third of a century we have used digitalis, and its several preparations in heart troubles, and though we have hoped for all things, and believed all things, even after repeated failures and disappointments from its use, that have been said and written of its value in cardiac affections, because we desired them to be true, candor compells us to say now, that it has proven one of the most, if not the most unsatisfactory remedies we have ever prescribed! We are aware that this is taking strong ground in the face of the testimony extant upon its value, and usefulness in diseases of the heart; and we would gladly record our experience on the popular side, and join in the eulogiums which have been written, and the pæns that have been sung for so long a period of time in praise of the "opium of the heart," could we do so with a proper regard for the truth as it has been made manifest in our experience! We are, therefore, reluctantly compelled to say that digitalis in our hands has rarely been attended with other than negative results in cardiac disease, even in cases where there could be no shadow of question as to a correct diagnosis.

In view of such an experience, and the growing and alarming frequency of heart troubles, it was perfectly natural that we should seek for more reliable and efficacious means of relief in such cases.

Careful clinical observation and experience in a number of well defined cardiac troubles, organic, and functional, with Night-blooming cereus, and Black cohosh, have established their value and efficacy in cases where digitalis had proven utterly valueless, or attended only with slight amelioration of the troubles. It would be obviously impracticable to enter into details and give the cases and results in a brief editorial notice, of the value of the above named articles, and while we would in no wise vaunt them as specifics in cardiac troubles, we would like to invite professional attention to their value and usefulness at this time, when so much more call is made for relief in that class of cases than formerly.

At a future time we may have more to say upon their administration and effects. The articles were used in the form of *fluid extract*, and from the reliable house of Sharp & Dohme, of this city. The dose of the *cactus grandiflora*, as used by us, is from two to ten drops three times a day or oftener; and that of *cimicifuga racemosa* from twenty to fifty drops at the same intervals. A tendency of the latter to produce violent headache, when given in the minimum, or even smaller dose, compelled is discontinuance in a few cases. But more anon.

